# Association of dietary patterns and hyperuricemia: a cross-sectional study of the Yi ethnic group in China

**DOI:** 10.29219/fnr.v62.1380

**Published:** 2018-04-25

**Authors:** Xirun Liu, Shanshan Huang, Wangdong Xu, Aijing Zhou, Hui Li, Rong Zhang, Ya Liu, Yan Yang, Hong Jia

**Affiliations:** 1 School of Public Health, Southwest Medical University, Luzhou, China; 2 Department of Medical Record, Sichuan University West China Hospital, Chengdu, China

**Keywords:** dietary pattern, hyperuricemia, factor analysis, cross-sectional study, the Yi ethnic group

## Abstract

**Background:**

Diet plays an important role in the development of hyperuricemia (HUA), but evidence for association between overall dietary patterns and HUA is scarce and inconsistent. The present study aims to explore association of dietary patterns and HUA among the Yi ethnic group of China.

**Methods:**

This is a cross-sectional study involving people aged more than 18 years. Principal component factor analysis (PCFA) on food groups from a semi-quantitative 52-item food frequency questionnaire was applied to identify dietary patterns. HUA status was regressed on tertiles of factor scores to estimate prevalence ratio (PR) by using log-binomial model.

**Results:**

Of the 1,893 participants (18–96 years), 398 (21.0%) were diagnosed with HUA. Three dietary patterns were identified: ‘plant-based’, ‘animal products’, and ‘mixed food’. The ‘animal products’ was characterized by high intake of fish, animal giblets, fresh meat, and wheat products. After adjustment for potential confounders, the highest tertile of ‘animal products’ pattern score was associated with higher prevalence of HUA when compared with the lowest tertile (PR: 1.34, 95% CI: 1.06–1.70). The other two patterns were not related to HUA.

**Conclusions:**

‘Animal products’ dietary pattern was correlated with HUA among the Yi ethnic group of China.

Hyperuricemia (HUA) is a metabolic disease caused by purine metabolic abnormalities. It is characterized by increased formation or reduced excretion of serum uric acid (SUA). Most patients with HUA are asymptomatic and do not receive treatment. However, previous studies indicated that HUA is an important risk factor for hypertension (1), diabetes (2), cardiovascular disease (CVD) (3), metabolic syndrome (MetS) (4), and that it also features in the pathophysiology of gout (5) and renal damage (6, 7). In recent decades, the prevalence of HUA has significantly increased, accompanied with younger age (8, 9). The prevalence of HUA was increasing rapidly in China as well (5, 10).

Genetics, lifestyle, and diet are susceptible factors contributing to HUA. It is accepted that diet is the most modifiable factor (11, 12). In nutritional epidemiology, researches are mainly paying attention to single nutrient or food (13) and do not consider the complexity of diet, and interactions of nutrients or foods. Representative of comprehensive dietary variables, dietary pattern attempts to reveal the influence of overall diet. Hence, it predicts association of nutrition and diseases more effectively, and adjusting a comprehensive dietary pattern is more effective than adjusting intake of single food or nutrient in the disease prevention and control. Dietary patterns analysis has been applied to evaluate association of diet with Mets (14, 15), CVD (16), diabetes (17), and obesity (18). However, there are few studies about dietary patterns and HUA. In addition, available evidence did not show consistent results that which pattern is protective or risky for HUA (19, 20).

Liangshan Yi Autonomous Prefecture is located in the southwestern Sichuan province, where the aboriginal Yi ethnic group lives a relatively primitive life and keeps its unique dietary culture and custom. The prevalence of HUA among adult Yi people was 14.71% in 2007 (21), while the prevalence in rural area was up to 22.0% in 2014 (22), significantly higher than other areas (5, 23). Diet is related to the incidence of HUA, but whether the diet of Yi people renders the health of its people a challenge remains to be clarified. Therefore, this study aims to characterize their dietary patterns and assess the correlation of these patterns with the prevalence of HUA among the Yi ethnic group of China.

## Methods

### Participants

This is a cross-sectional study based on Yi people aged more than 18 years. A representative sample of the Yi ethnic group was obtained by a multistage stratified cluster sampling method. Details of survey methods and sample selection are described in our previous study (24). Of the 1,918 participants aged 18–96 years, 1,893 (98.70%) were included for the analyses. Twenty-five of the participants were excluded because of no SUA measurement or more than 50% of the dietary questions being incomplete. Exclusion criteria were (1) renal failure, (2) medication for HUA or gout, (3) pregnancy, and (4) inability to communicate or walk normally. The corresponding protocol was approved by the Ethics of Research Committee of the Southwest Medical University. Informed consent was obtained from all participants.

### Data collection

Data collection was accomplished by local medical workers, well-trained investigators, and field workers. Details of the procedure are as follows:

#### Questionnaire survey

Data collection was performed by face-to-face interviews with interviewer-administered questionnaires. Information on socio-demographic characteristics and other potential confounders such as age, gender, years of education (≤6 years, 7–12 years, ≥13 years), drinking and smoking status (yes or no) and presence of hypertension was collected using a structured questionnaire. A semi-quantitative 52-item food frequency questionnaire (FFQ) was used to measure habitual diet over the past year. The FFQ included questions on the types of food items, frequency (daily, weekly, monthly, yearly, or never), and amount (portion size in grams per time) of food consumption. Total alcohol consumption was the sum of daily intake of different alcoholic beverages. Consumption of cooking oil of a family was recorded and averaged for assessing the individual’s cooking oil consumption per day. The FFQ was designed based on culture-specific dishes, recipes, and tested on a local sample to check for clarity and applicability under the Yi culture.

#### Physical examination

Heights were measured to the nearest 0.1 cm using a secure standard height measuring equipment while wearing no shoes. Weight was collected using a calibrated electronic digital scale, accurate to 0.1 kg with subjects wearing only light underwear. Body mass index (BMI) was calculated by weight (kg)/height^2^ (m^2^). Waist circumference (WC) was measured with a tape measure to the nearest 0.1 cm around the midpoint between the lowest rib and the iliac. Hip circumference was measured to the nearest 0.1 cm with the soft tape placed around the symphysis pubis and the posterior gluteus maximus. Waist–hip ratio (WHR) was calculated by WC (cm)/hip circumference (cm). Blood pressure (BP) was measured by electronic sphygmomanometer after participant rested for 5 min.

#### Laboratory test

Fasting blood samples were collected from each individual and sent to Liangshan center for disease control and prevention. SUA, fasting plasma glucose (GLU), triglycerides (TG), total cholesterol (TC), high-density lipoprotein cholesterol (HDL-C), and low-density lipoprotein cholesterol (LDL-C) were measured by an automatic biochemical analyzer (Mindary, BS-820, Shenzhen, China).

### Definition of disease

HUA was diagnosed when SUA ≥420 μmol/L (7.0 mg/dL) for men, and SUA ≥360 μmol/L (6.0 mg/dL) for women (25). Hypertension: BP ≥140/90 mmHg, or usage of antihypertensive drugs. Hyperlipemia: if one or more of the following criteria were satisfied: (1) TC >5.72 mmol/L, (2) TG >1.70 mmol/L, and (3) HDL-C <1.3 mmol/L in women and <1.0 mmol/L in men (26).

### Assessment of dietary intake

Food items were categorized into 18 food groups based on Food Composition Table, similarities in ingredients, and purine contents (Supplementary Table 1). The total intake of food group depended on summing the daily gram intake of each food item within the group. Principal component factor analysis (PCFA) with varimax orthogonal rotation was applied. The number of patterns was determined by the cumulative contribution rate of variance, eigenvalue >1, and the interpretability of the results. Food groups with rotated factor loading greater than 0.35 were regarded as main components of each pattern. Dietary pattern scores were calculated by summing the product of standardized intake of each group multiplied by regression coefficients and were categorized into tertiles.

**Table 1:** Demographics, lifestyle characteristics, anthropometric measurements, and serum biochemical_indexes of Yi People participants in the nutrition survey (*n* = 1,893)

Variables	Participants with HUA	Participants without HUA	*p*
	
	*n* = 398	*n* = 1,495	
Gender			<0.001
Male	270 (67.8)	636 (42.5)	
Female	128 (32.2)	859 (57.5)	
Age group			0.247
18–44	244 (61.3)	847 (56.7)	
45–59	98 (24.6)	415 (27.7)	
≥60	56 (14.1)	233 (15.6)	
Years of education			<0.001
≤6	227 (57.0)	1,162 (77.7)	
7–12	138 (34.7)	274 (18.3)	
≥13	33 (8.3)	59 (3.9)	
Smoking			<0.001
Yes	170 (42.7)	486 (32.5)	
No	228 (57.3)	1,009 (67.5)	
Drinking			<0.001
Yes	231 (58.0)	723 (48.4)	
No	167 (42.0)	772 (51.6)	
BMI (kg/m^2^)	23.58 ± 3.57	22.25 ± 3.16	<0.001
Waistline	83.46 ± 10.61	78.66 ± 9.43	<0.001
WHR	0.88 ± 0.07	0.86 ± 0.07	<0.001
SBP (mmHg)	131.95 ± 18.96	125.59 ± 17.68	<0.001
DBP (mmHg)	79.86 ± 13.78	76.09 ± 12.23	<0.001
GLU (mmol/L)	5.57 ± 1.46	5.59 ± 1.66	0.819
TC (mmol/L)	5.13 ± 1.08	4.89 ± 0.97	<0.001
TG (mmol/L)	1.79 ± 1.27	1.37 ± 1.13	<0.001

Arithmetic mean values and standard deviations (SD) for continuous variables; number of participants and percentages for categorical variables; test for differences in the characteristics of the participants with and without HUA were taken using the Chi-square test for categorical variables and the ANOVA analyses for continuous variables.

BMI: body mass index = weight (kg)/height^2^ (m^2^); WHR: waist–hip ratio = waistline (cm)/hipline (cm); SBP: systolic blood pressure; DBP: diastolic blood pressure; SUA: serum uric acid; HUA: hyperuricemia; GLU: glucose; TC: serum cholesterol; TG: serum triglycerides.

### Statistical analysis

Frequencies (%), means ± standard deviations (SD) were used to describe various socio-demographic characteristics, lifestyle, anthropometric measurement, and clinical characteristics of participants in the nutrition survey. Binary HUA status was regressed on tertiles of factor scores to obtain prevalence ratios (PRs) by using log-binomial model. All the analyses were conducted using the SAS 9.3 software and two-tailed and considered statistically significant when *p* < 0.05.

## Results

### Characteristics of participants

A total of 1,893 participants were included, and the prevalence of HUA was 21.0%. Characteristics of this population were listed in Table 1. Participants with and without HUA showed significant differences in terms of gender, education, smoking, and drinking status. Compared with those without HUA, participants with HUA showed higher expression of TG, TC, and SUA, and higher BMI, WC, WHR, and BP.

### Extraction of dietary patterns

We identified three dietary patterns in this population, which we named: a ‘plant-based’ dietary pattern, as it was characterized by high intake of vegetables, fruits, mushroom, algae food, legumes, nuts, brawn, and bacon; an ‘animal products’ dietary pattern, which was characterized by high intake of fish, animal giblets, fresh meat, wheat products; and a ‘mixed food’ dietary pattern, as it was mostly composed of rice, cereal, tubers, snack, dessert, eggs, and animal giblets (Table 2). The three patterns explained 34.22% of total variance, with the ‘plant-based’, ‘animal products’, and mixed food’ constituting 12.27%, 11.48%, and 10.47%, respectively, of the total variance. Food intake across tertiles of each dietary pattern was listed (Supplementary Table 2).

**Table 2:** Dietary pattern factor loading matrix of the Yi people

Food groups	Plant-based	Animal products	Mixed food
Mushroom and algae food	0.672^a^	-	-
Vegetables	0.613	-	-
Legumes	0.518	-	0.365
Nuts	0.506	-	-
Brawn, bacon	0.486	-	-
Fruits	0.372	-	-
Sugary beverages	-	-	-
Dairy	-	-	-
Oil	-	-	-
Alcoholic beverages^b^	-	-	-
Wheat and its products	-	0.856	-
Fish	-	0.847	-
Fresh meat	0.357	0.472	-
Snacks and dessert	-	-	0.624
Animal giblets	-	0.458	0.612
Other cereal and tubers	-	-	0.574
Eggs	-	-	0.458
Rice	-	-	0.409
Contribution rate (%)	12.27	11.48	10.47
The cumulative contribution rate (%)	12.27	23.75	34.22

a Factor loading > 0.35 are listed.

b Gram or Liang.

### Association between dietary patterns and HUA

Higher adherence to ‘animal products’ pattern was related to higher prevalence of HUA (crude model: *p*-trend = 0.027). After adjustment for confounders, the association between this pattern and HUA was still significant. (the highest tertile: PR = 1.34; 95% CI: 1.06–1.70; *p*-trend = 0.031). The other two patterns, ‘plant-based’ and ‘mixed food’, were not associated with HUA (Table 3).

**Table 3:** Analysis of the dietary patterns and HUA of the Yi people

Dietary patterns	Participants with HUA	SUA (mmol/L)	Crude model		Adjusted model	
			
	*n* (%)	Median	PR (95% CI)	*p* for trend	PR (95% CI)	*p* for trend
Plant-based				0.750		0.763
T1	124 (19.7)	316.0	Reference		Reference
T2	121 (19.2)	323.0	0.94 (0.75–1.18)		0.93 (0.75–1.15)	
T3	153 (24.2)	340.0	1.15 (0.93–1.43)		1.03 (0.84–1.26)	
Animal products				0.027		0.031
T1	97 (15.4)	310.0	Reference		Reference	
T2	130 (20.6)	313.0	1.32 (1.03–1.69)*		1.32 (1.04–1.68)*	
T3	171 (27.1)	354.0	1.69 (1.34–2.14)*		1.34 (1.06–1.70)*	
Mixed food				0.157		0.221
T1	156 (24.7)	334.0	Reference		Reference	
T2	124 (19.7)	326.0	0.85 (0.69–1.05)		0.86 (0.71–1.05)	
T3	118 (18.7)	318.0	0.90 (0.71–1.12)		0.97 (0.78–1.20)	

Crude Model: No adjustment for any confounders.

Adjusted Model: Adjustment for age group (18–44, 45–59, 60 years); gender (male/female); BMI: body mass index; smoking (smoker/non-smoker); drinking (drinking/no drinking); hypertension (with/without); hyperlipidemia (with/without).

The median value of each tertile was assigned to each subject in the same tertile and treated as a continuous variable in regression analysis.

* *p* < 0.05.

## Discussion

In this study, three major dietary patterns, ‘plant-based’, ‘animal products’, and ‘mixed food’ were identified. The ‘animal products’ pattern was positively correlated with HUA. However, the other two patterns showed no association with HUA.

The ‘animal products’ pattern was characterized by high intake of fish, fresh meat, animal giblets, and legumes. To date, several studies have discussed the association of diet and HUA. The ‘animal product and fried food’ pattern, which indicated high consumption of pork, eggs, animal, poultry, and fried wheat, was associated with HUA (27). Tsai et al. reported that uric acid–prone pattern, including seafood, meat, beverages, organ meat, eggs, was positively related to concentration of SUA (19). Zykova et al. reported that high intake of meat was associated with elevated SUA, and intake of fish was not associated with high levels of SUA (13). Some other studies indicated that high intake of red meat, animal organ, and seafood are related to elevated levels of SUA and an increased risk of gout, because these food were purine-rich foods, and excessive purine intake may increase the levels of SUA (28, 29) and cause HUA (30, 31). In addition, high consumption of animal products, especially red meat, was often accompanied with excessive intake of calorie and fat, which may lead to obesity or centripetal obesity. HUA was associated with obesity (29, 32), and the incidence of HUA was increased accompanied with elevated BMI (29). Besides, centripetal obesity was a powerful stimulus for increasing the plasma insulin level and, therefore, it may increase the risk of HUA (33). In this study, we found that BMI of participants in the HUA group was significantly higher than that of the non-HUA group, but there was no significant difference in BMI among people from different dietary patterns, possibly suggesting that the high prevalence of HUA in the Yi people was mainly attributed to their diet. Collectively, our findings indicated that the prevalence of HUA was related to the ‘animal products’ pattern.

The ‘plant-based’ pattern was characterized by high intake of vegetables, fruits, legumes, nuts, brawn, and bacon. Fruits and vegetables are rich in dietary fiber, vitamin C, and folate, which were considered as protective factors for HUA (13, 34). On the contrary, some other studies did not suggest fruits and vegetables as protective factors (30, 35). Zhang et al. showed that fruit pattern, including soybean products, fruits, vegetables, and starchy tubers, was negatively related to HUA (27). A study among the Taiwanese indicated that vegetable and fruit pattern was not related to SUA concentration (20). High intake of vitamin C may reduce the SUA levels, owing to uricosuric effects mediated by inhibition of uric acid transporters 1 (URAT1) and/or sodium-dependent anion cotransport (36, 37). Fiber is possible to bind to SUA. One-third of the SUA produced every day is eliminated in intestinal secretions and saliva (27, 38). Our findings showed that the ‘plant-based’ pattern was not associated with HUA, where fruits and vegetables may decrease the risk of HUA. It is noteworthy that brawn and bacon heavily consumed in this pattern contributes to elevated SUA, increasing the risk of HUA (28, 30). Therefore, it is possible that the influence of purine and fat-rich foods may counteract the protective effects that fruits and vegetables provided for HUA, which may reflect the superiority of application of dietary patterns analysis.

‘Mixed food’ pattern, comprising rice, cereal, tubers, snack, dessert, eggs and animal giblets, was not associated with HUA. Cereal is an important source of vitamin B and is rich in carbohydrates, which are inversely correlated with levels of SUA (13, 34). High consumption of animal giblets was regarded as an adverse factor, resulting in elevated levels of SUA (31, 34). High consumption of snack and dessert contributed to overweight and upregulation of SUA levels (33). Studies about the association of SUA/HUA and animal protein were inconsistent. Zykova et al. reported that the high consumption of egg was associated with elevated levels of SUA (13). By contrast, Choi et al. showed that a higher intake of animal protein was not associated with an increased risk of gout (31), and consumption of animal protein was associated with higher prevalence of HUA (13). The overall effect of ‘mixed food’ pattern could be explained by the interaction of foods or nutrients. Further studies are needed to give more information about the interaction effects and confirm the result.

The present study has several strengths. First, questionnaires were administered by well-trained investigators and were not self-completed. This may lead to higher response rate, and provide convenience that illiterate people could also be included in the investigation. Second, collected dietary data over the past year could capture the picture of long-term dietary behaviors for individuals. Third, this study identified healthy or unhealthy dietary patterns and could form the starting point for development of new dietary guideline(s).

This study has several limitations. First, this is a cross-sectional study, which may limit the ability to make a causal inference. Second, the sample size may not be sufficient enough; therefore, larger sample would be considered in further research.

## Conclusion

The study showed that ‘animal products’ pattern, characterized by high intake of fish, fresh meat, animal giblets, and wheat products, was correlated with increased prevalence of HUA in the Yi ethnic group of China. Our findings may aid in developing dietary guidelines for adults and providing the population with guidance for healthy dietary patterns based on the access to local culture and customs, and encourage people in the risky pattern to change their dietary structure to prevent and control HUA.

## Supplementary Material

Association of dietary patterns and hyperuricemia: a cross-sectional study of the Yi ethnic group in China
