# Supplementary material for: Association of dietary patterns and hyperuricemia: a cross-sectional study of the Yi ethnic group in China
Source: Food Nutr Res. 2018 Apr 25;62:10.29219/fnr.v62.1380. doi: 10.29219/fnr.v62.1380 (PMC5917417; doi:10.29219/fnr.v62.1380)
Supplement: Association of dietary patterns and hyperuricemia: a cross-sectional study of the Yi ethnic group in China [file FNR-62-1380-s001.pdf]

Supplementary material 1

Table 1. 18 food groups used in the analysis

Food groups	Components
Wheat and its products	Wheat, bread, noodles
Rice	Rice and its products
Other cereals and tuber	Corn, millet, sorghum, potato, taro, sweet potato
Legumes	Soybean, bean curd, white kidney bean, bean milk
Vegetables	Potato, tomato, eggplant, dark green and yellow vegetable, cauliflower
Mushroom and algae food	Edible fungus, mushrooms, seaweed, kelp
Fruits	Deep yellow orange fruits, bananas, apples, strawberries, citrus fruits, grapes
Nuts	Nuts and its products
Fresh meat[a]	Red meat, poultry meat
Brawn, bacon	Brawn, bacon, salted and smoked meat
Animal giblet	Animal giblet
Fish	Fish, seafood, shrimp, crab, shellfish (From deep sea or freshwater)
Dairy	Milk and dairy products
Eggs	Eggs, duck eggs, goose egg
Snacks and dessert	Biscuits, cakes, fritters
Sugary beverages	Carbonated drinks and juices
Alcoholic beverages	Alcoholic beverages
Oil	Vegetable oil, animal fat

[a] Most of the meat they consumption is red meat.

Table 2. Demographic, lifestyle characteristics, anthropometric measurements and serum biochemical indexes of Yi People participants in the nutrition survey. (n = 1893)

Variables	Participants With HUA n=398	Participants Without HUA n=1495	*P* value
Gender			<0.001
Male	270(67.8)	636(42.5)	
Female	128(32.2)	859(57.5)	
Age group			0.247
18-44	244(61.3)	847(56.7)	
45-59	98(24.6)	415(27.7)	
≥60	56(14.1)	233(15.6)	
Years of education			<0.001
≤6	227(57.0)	1,162(77.7)	
7-12	138(34.7)	274(18.3)	
≥13	33(8.3)	59(3.9)	
Smoking			<0.001
Yes	170(42.7)	486(32.5)	
No	228(57.3)	1,009(67.5)	
Drinking			<0.001
Yes	231(58.0)	723(48.4)	
No	167(42.0)	772(51.6)	
BMI (kg/m^2)	23.58±3.57	22.25±3.16	<0.001
Waistline	83.46±10.61	78.66±9.43	<0.001
WHR	0.88±0.07	0.86±0.07	<0.001
SBP (mmHg)	131.95±18.96	125.59±17.68	<0.001
DBP (mmHg)	79.86±13.78	76.09±12.23	<0.001
GLU (mmol/L)	5.57±1.46	5.59±1.66	0.819
TC (mmol/L)	5.13±1.08	4.89±0.97	<0.001
TG (mmol/L)	1.79±1.27	1.37±1.13	<0.001

a. Arithmetic mean values and standard deviations(SD) for continuous variables; number of participants and percentages for categorical variables; Test for differences in the characteristics of the participants with and without HUA were taken by using the Chi-square test for categorical variables and the ANOVA analyses for continuous variables.
b. BMI: Body mass index = weight (kg)/height^2 (m^2); WHR: waist-hip ratio = waistline (cm)/ hipline (cm); SBP: systolic blood pressure; DBP: diastolic blood pressure; SUA: Serum uric acid; HUA: hyperuricemia; GLU: glucose; TC: serum cholesterol; TG: serum triglycerides.

Table 3. Dietary pattern factor loading matrix of the Yi people

Food groups	Plant-based	Animal products	Mixed food
Mushroom and algae food	0.672[a]	-	-
Vegetables	0.613	-	-
Legumes	0.518	-	0.365
Nuts	0.506	-	-
Brawn, bacon	0.486	-	-
Fruits	0.372	-	-
Sugary beverages	-	-	-
Dairy	-	-	-
Oil	-	-	-
Alcoholic beverages[b]	-	-	-
Wheat and its products	-	0.856	-
Fish	-	0.847	-
Fresh meat	0.357	0.472	-
Snacks and dessert	-	-	0.624
Animal giblets	-	0.458	0.612
Other cereal and tubers	-	-	0.574
Eggs	-	-	0.458
Rice	-	-	0.409
Contribution rate (%)	12.27	11.48	10.47
The cumulative contribution rate (%)	12.27	23.75	34.21

[a]Factor loading ≥ 0.35 are listed
[b]Grams of alcohol

Supplementary material: 2 Table 4.
(See in document: Supplementary material: 2)

Table 5. Analysis of the dietary patterns and HUA of the Yi people

Dietary patterns	Participants with HUA	SUA (mmol/L)	Crude Model		Adjusted Model	
	n (%)	median	PR(95%CI)	*P* for trend	PR(95%CI)	*P* for trend
**Plant-based**						
T1	124(19.7)	316.0	Reference	0.750	Reference	0.763
T2	121(19.2)	323.0	0.94(0.75-1.18)		0.93(0.75-1.15)	
T3	153(24.2)	340.0	1.15(0.93-1.43)		1.03(0.84-1.26)	
**Animal products**				0.027		0.031
T1	97(15.4)	310.0	Reference		Reference	
T2	130(20.6)	313.0	1.32(1.03-1.69)*		1.32(1.04-1.68)*	
T3	171(27.1)	354.0	1.69(1.34-2.14)*		1.34(1.06-1.70)*	
**Mixed food**				0.157		0.221
T1	156(24.7)	334.0	Reference		Reference	
T2	124(19.7)	326.0	0.85(0.69-1.05)		0.86(0.71-1.05)	
T3	118(18.7)	318.0	0.90(0.71-1.12)		0.97(0.78-1.20)	

Crude Model : Do not adjustment for any confounders.
Adjusted Model : Adjustment for age group (18-44, 45-59, 60 years), gender (male/female); BMI: body mass index, smoking (smoker/non-smoker), drinking (drinking /no drinking) , hypertension (with/without), hyperlipidaemia (with/without).
The median value of each tertile was assigned to each subject in the same tertile and treated as a continuous variable in regression analysis.
* $p < 0.05$
